# Using virtual reality in medical education to teach empathy

**DOI:** 10.5195/jmla.2018.518

**Published:** 2018-10-01

**Authors:** Elizabeth Dyer, Barbara J. Swartzlander, Marilyn R. Gugliucci

**Affiliations:** Associate Dean for Library Services and Research and Teaching Librarian, Abplanalp Library, University of New England, Portland, ME 04103; Research and Teaching Librarian, Ketchum Library, University of New England, Biddeford, ME 04005; Professor and Director of Geriatrics Education Research, Division of Geriatric Medicine, College of Osteopathic Medicine, University of New England, Biddeford ME 04005

## Abstract

**Objective:**

The project adopted technology that teaches medical and other health professions students to be empathetic with older adults, through virtual reality (VR) software that allows them to simulate being a patient with age-related diseases, and to familiarize medical students with information resources related to the health of older adults.

**Methods:**

The project uses an application that creates immersive VR experiences for training of the workforce for aging services. Users experience age-related conditions such as macular degeneration and high-frequency hearing loss from the patient’s perspective. Librarians and faculty partner to integrate the experience into the curriculum, and students go to the library at their convenience to do the VR assignment.

**Results:**

The project successfully introduced an innovative new teaching modality to the medical, physician assistant, physical therapy, and nursing curricula. Results show that VR enhanced students’ understanding of age-related health problems and increased their empathy for older adults with vision and hearing loss or Alzheimer’s disease.

**Conclusion:**

VR immersion training is an effective teaching method to help medical and health professions students develop empathy and is a budding area for library partnerships. As the technology becomes more affordable and accessible, it is important to develop best practices for using VR in the library.

The University of New England (UNE) is in the third year of an educational project that uses virtual reality (VR) technology to teach empathy to medical and other health professions students. Funded by the National Network of Libraries of Medicine New England Region, the project also familiarizes students with National Library of Medicine information resources related to older adult health. UNE research and teaching librarians collaborate with faculty from the UNE College of Osteopathic Medicine on the Biddeford campus and the Westbrook College of Health Professions on the Portland campus to implement the project. To date, more than 600 students from the programs in medicine, physician assistant, physical therapy, and nursing have participated.

The project uses software from Embodied Labs (EL), a new company that creates VR labs for training the workforce for aging services. The application is unique in that it puts the learner in the shoes of the patient to teach about the aging experience from a first-person perspective. The “Alfred Lab” teaches about macular degeneration and hearing loss from the perspective of a seventy-four-year-old African American man. Other new labs teach about Alzheimer’s disease and end-of-life conversations. UNE was the first institution to license this novel product and has been on the forefront of testing each module as it is developed and implementing use with different student populations.

The planning phase required purchasing equipment, developing the pre- and post-assessments that are embedded in the software, creating the assignment to integrate into the geriatrics curriculum, and training staff. The authors tested the project with student library workers as well as volunteer medical students. First-year implementation involved 178 first-year medical students who had a 10-week window to complete the assignment at the library. The 24-hour library schedule provided many opportunities for students to complete the work at a time that was convenient for them. The second project year expanded the pilot project to include first-year physician assistant students. While not part of the planned project, other programs also participated, due to the great interest among faculty and students.

Initial grant funding enabled the purchase of 2 VR kits for each campus. Each kit includes an Alienware laptop, Oculus Rift headset and sensor, and Leap Motion hand-tracking device with mount and long USB cable. Optional accessories include Pelican cases for storage and transport, cable sleeve, and cleaning wipes. We originally intended students to check out a kit for in-library use, but we opted to carve out space for ready-to-go stations to make the experience as easy as possible (Figure 1). Second-year funding allowed for 2 more kits, and we chose desktop stations to see if the technology ran smoother. Early experiences showed glitchy connections between the laptop and headset, but recent experiences show improvement and we saw no difference in laptop versus desktop function. Depending on computer configurations, each kit currently runs from about $2,000–$2,500, with some item costs trending down.

**Figure 1:**
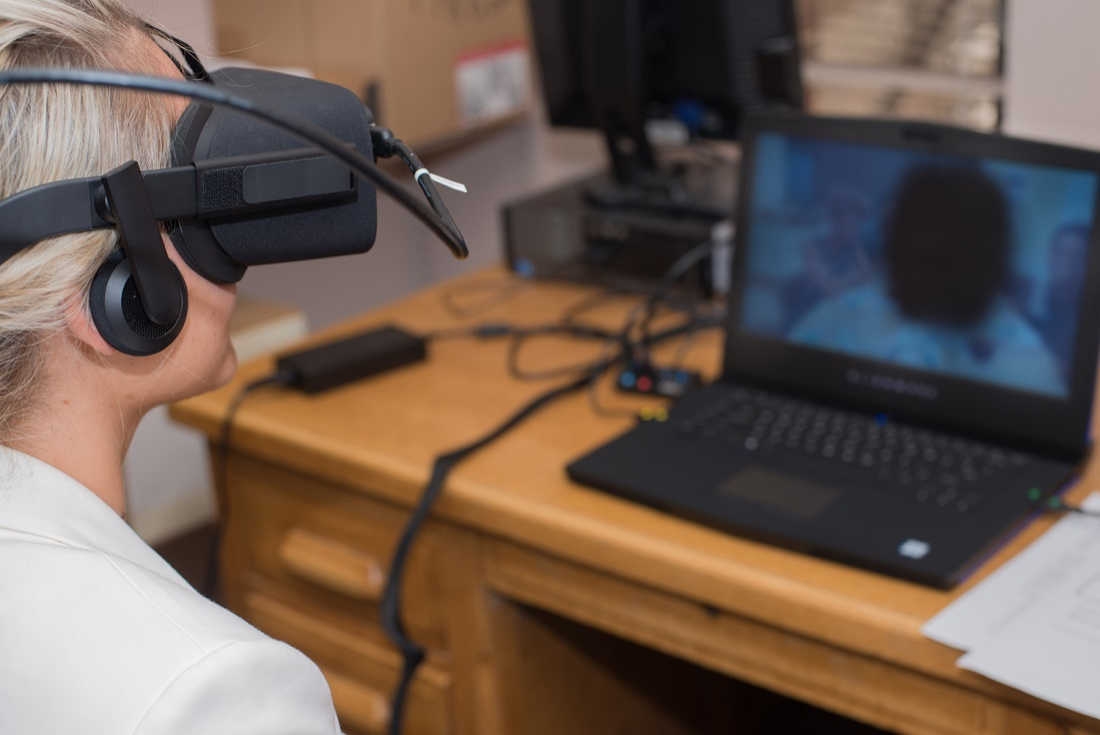
University of New England virtual reality station

Software was downloaded from EL and updated as necessary. Pre- and post-assessments, initially created in Google Forms, were migrated to REDCap for better control and security of data. Faculty and library staff codeveloped assessments unique to each program. EL accommodated our requests for program-specific login codes, which were linked to program-specific assessments. This enabled easy separation of data by program in REDCap.

Some limitations of the project include staff time and space issues. Implementation became a time-management challenge for library staff who juggled multiple programs and time frames. The stations require secure space in the libraries to protect the equipment when not in use but that is accessible to students when needed. Library staff also need to have ready access to information technology staff who can help maintain and troubleshoot the technology when problems arise. A valid assessment tool needs to show that the intervention successfully improves student learning and empathy.

Best practices for using VR in education need to be developed. For example, some users experience dizziness or nausea using VR. EL recommends that time with the headset be limited to about ten minutes. The “Alfred Lab” fits in this time frame, but newer modules are longer and broken into parts. Busy students may forego breaks between parts and then complain of adverse effects. A challenge to the project has been keeping the headsets sanitary between users. We used alcohol-free wipes on the foam frames that touch the face, but it is unclear how this affects the integrity of the foam. Oculus now offers protective liners that can be wiped, which we plan to use this year on all of our headsets. The upcoming third year of the project involves refining logistics to reflect best practices, testing the newest module against the two that have already been tried, and using a validated assessment tool to evaluate the efficacy of the educational experience.

The technology has been well received by UNE students and faculty, and has increased the use and value of UNE Library Services. Students provide positive feedback about the incorporation of a new technology tool into their learning. The project has fostered new faculty-librarian collaborations and brought students into the library who otherwise might not have come. Assessment results indicate that students demonstrate increased understanding of and empathy with older adults who have age-related conditions such as macular degeneration and hearing loss. Research indicates that empathy leads to better patient care and outcomes and that educational interventions work [1], so it is worthwhile to address this subject area in the curriculum. VR immersion training is an effective teaching method to help develop empathy and is a budding area for library partnerships.
